# Participation of the purinergic P2X7 receptor in molecular complexes in the nucleus of human chondrocytes

**DOI:** 10.1007/s00018-026-06274-2

**Published:** 2026-06-17

**Authors:** Elisabetta Lambertini, Letizia Penolazzi, Anna Chierici, Riccardo Nadalini, Chiara Sief, Francesca Begnozzi, Massimo Bonora, Paolo Pinton, Chiara Bianchini, Francesco di Virgilio, Roberta Piva

**Affiliations:** 1 Laboratorio centralizzato di ricerca preclinica, University of Ferrara, Ferrara, Italy; 2 Department of Neuroscience and Rehabilitation, University of Ferrara, Ferrara, Italy; 3 Department of Medical Sciences, Section of Experimental Medicine, University of Ferrara, Ferrara, Italy; 4 Maria Cecilia Hospital, GVM Care & Research, Cotignola, RA 48033 Italy

**Keywords:** purinergic P2X7 receptor, Human chondrocytes, Lamin A/C, Emerin, SUN2

## Abstract

In addition to the purinergic receptor P2X7R’s known activity as a sensor of damage-associated molecular patterns (DAMPs), evidences support its role in maintaining tissue homeostasis. Its presence in cellular compartments other than its usual transmembrane localization suggests its involvement in specific signaling pathways. This study aimed to analyze P2X7R in the nucleus of human chondrocytes and search for potential interacting partners. Through co-immunoprecipitation and proximity ligation assay we discovered that, independent of extracellular ATP levels, P2X7R is abundantly present in both the nuclear membrane and in the nucleoplasm, where it is found in close proximity to lamin A/C (a component of the nuclear lamina), emerin (a protein involved in the assembly and disassembly of the nuclear envelope), and SUN2 (an inner nuclear membrane protein that facilitates the transmission of mechanical forces). Furthermore, chromatin immunoprecipitation revealed the participation of P2X7R in molecular complexes located in the promoter of specific genes including Sox9, TRPS1, FOXO3a, integrin β2 and connective tissue growth factor. Overall, this evidence reveals for the first time novel partners of P2X7R that place it in an intricate network that influences nuclear structure, mechanosensitivity, chromatin organization, and gene expression. Specifically, on the one hand, a close association between P2X7R and nuclear proteins participating in the LINC (Linker of Nucleoskeleton and Cytoskeleton) complex (lamin A/C, emerin, and SUN2) places it among the factors involved in mechanosignaling and the maintenance of nuclear integrity; on the other, its recruitment to specific gene promoters suggests that it may act as a transcription regulator.

**Supplementary Information:**

The online version contains supplementary material available at https://doi.org/10.1007/s00018-026-06274-2.

## Introduction

P2X7R is a non-selective cationic transmembrane purinergic receptor present in almost all tissues and organs, where it mediates specific responses by acting as a sensor for damage-associated molecular patterns (DAMPs), specifically extracellular ATP (eATP) [1–3]. Following membrane damage or necrosis, mechanical stress, inflammation and hypoxia all cells can potentially release ATP into the extracellular environment, which can then act in an autocrine/paracrine manner to influence local purinergic signaling: this is the scenario that traditionally explains the role of the P2X7R, activated precisely by high concentrations of eATP [4, 5]. However, there is evidence supporting a role of the P2X7R also in maintaining tissue homeostatic balance in response to a wide range of physiological stimuli. This may occur when positive allosteric modulators of the receptor increase its sensitivity to low levels of eATP, explaining the relevance of P2X7R in undamaged tissues [6]. This phenomenon occurs in proximity to finely tuned ATP-releasing structures that through specific transduction pathways regulate physiological cellular responses [7, 8]. Another important aspect to consider in explaining the potential role of P2X7R in physiological contexts of various cell-types is its presence in the intracellular compartments including the endoplasmic reticulum, lysosomes, phagosomes, mitochondria, and even the nucleus [9–13]. This receptor trafficking can be explained by the steps required before P2X7R reaches the appropriate sites, or by the degradation or recycling processes of the receptor itself. But there are many other implications to consider, such as the modulation of the receptor activity and its interactions with other molecules necessary to mediate potential non-canonical signaling. This suggests that P2X7R could be involved in novel signaling pathways at sites other than the plasma membrane, such as mitochondria and nucleus. Factors influencing P2X7R trafficking are poorly studied to date and may include post-translational modifications, interactions with specific protein partners, and the presence of stimuli that influence changes in the cellular environment [7, 12, 14].

For some years we have been interested in attributing a physiological role to P2X7R in cells of the skeletal system. We previously demonstrated the presence of P2X7R in the nucleus of osteoblasts, chondrocytes, and intervertebral disc cells, which are chondrocyte-like cells [10]. We now focus here on the significance of P2X7R in the nucleus of normal human chondrocytes and the potential involvement of this receptor in nuclear activities related to joint function.

Chondrocytes, the metabolically active cells within cartilage tissue, release ATP in a controlled manner, both under basal conditions and in response to mechanical stimuli [15–18]. They are essential to cartilage’s role in providing cushioning, flexibility, and support to joints and other parts of the body.

Evidence to date regarding the role of P2X7R in chondrocytes and chondrogenic differentiation after activation by eATP is limited and sometimes contradictory. There is evidence for its positive influence on chondrocyte survival, cartilage development and repair, but also cartilage degradation under certain conditions such as osteoarthritis [17, 19–21]. In any case these data refer to the membrane-bound P2X7R. In this context, the aim of the present study was to understand the potential involvement of P2X7R in nuclear signaling pathways, searching for possible partners participating in the intricate network of interactions between nucleus, cytoskeleton, and extracellular matrix (ECM) which influence nuclear structure, mechanosensitivity, chromatin organization, transcription factor activity regulation and, finally, the expression of specific genes [22–24]. The hypothesis has not been investigated before. We focused on possible association between P2X7R and lamin A/C (a component of the nuclear lamina, a network of intermediate filaments) [25], emerin (an inner nuclear membrane protein, involved in the assembly and disassembly of the nuclear envelope) [26], and SUN2 (a transmembrane protein of the inner nuclear membrane that facilitates the transmission of mechanical forces) [27]. These proteins participate in the LINC (Linker of Nucleoskeleton and Cytoskeleton) complex, which bridges the nuclear envelope and the cytoskeleton, facilitating communication and force transmission between these two cellular compartments [23, 28]. We focused on human primary chondrocytes, as cells whose functionality is profoundly influenced by the complex signaling network mentioned above. In order to analyze protein-protein interactions and understanding the composition of protein complexes co-immunoprecipitation (Co-IP) and Proximity Ligation Assay (PLA) was performed. Furthermore, the presence of P2X7R in complexes regulating gene expression such as the Lamina-Associated Domains (LADs) [29, 30] in the genome was evaluated by chromatin immunoprecipitation (ChIP). With this approach we have demonstrated for the first time a close association between P2X7R and molecular mechanisms related to the maintenance of nuclear structure and the regulation of gene expression, discovering new elements that could explain many more roles of P2X7R than those described so far.

## Materials and methods

### Reagents

General laboratory reagents were of analytical grade and were purchased from, Merck KgaA (MA, USA). Protease inhibitor cocktail, Magna ChIP Protein A + G magnetic beads (#16–663), Immobilon Western Chemiluminescent HRP substrate (# WBKLS0500), Protein A- 20 nm Colloidal Gold Labeled (#P6855), Bradford reagent (#B6916), Corning ^TM^ ITS+Premix (#354352) were also purchased from Merck KgaA. DMEM HG, Ham’s F12, FCS, L-glutamine, antibiotics (penicillin and streptomycin), and 1X phosphate-buffered saline (PBS) were obtained from Euroclone S.p.A (Milan, Italy). iTaq Universal Sybr Supermix and qPCR (quantitative polymerase chain reaction) plates were obtained from Bio-Rad (CA, USA). Lipofectamine 2000, ProLong Gold Antifade Mountant with DNA stain DAPI (4′, 6-diamidino-2-phenylindole; #P36935), TaqMan Assays and TaqMan™ Universal Master Mix II, were purchased from Thermo Fisher Scientific (Waltham, MA, USA). Hoechst 33258 (#94403) was obtained from Merck. eGFP-hP2X7R expression vector was obtained from Origene (Rockville, MD, USA). TGF-β3 was purchased from Mylteny Biotec (Bergisch, Gladbach, Germany). Antibodies used in this study are listed in Supplementary Table 1.

### Cell isolation and cultures

Human chondrocytes were isolated from nasal septum cartilage collected during septoplasty surgeries, after obtaining informed consent and approval of the Ethics Committee of the University of Ferrara and S. Anna Hospital (approval No. 2025 − 406). Briefly, cartilage fragments were minced into small pieces and rapidly incubated with 1 mg/mL type IV collagenase at 37 °C for 16 h [10]. Cells were harvested by centrifugation and plated (passage zero, P0) at a density of 2 × 10^4^ cells/cm^2^ in tissue culture flasks (25 cm^2^) or 24-well culture slides in standard medium (50% DMEM high-glucose/50% DMEM F-12/10% fetal calf serum) supplemented with antibiotics (penicillin 100 mg/mL and streptomycin 10 mg/mL), at 37 °C in a humidified atmosphere of 5% CO_2_. The cells were then expanded until confluent, harvested and used for the experiments here described (passage 2 to passage 4). For the induction of chondrogenesis, 5 × 10^5^ chondrocytes were seeded in 15 mL polypropylene conical tube (Corning, NY, USA) and centrifuged for 5 min at 500 g to form a 3D pellet. The supernatant was removed and replaced with chondrogenic medium (DMEM High Glucose supplemented with ITS+Premix: 6.25 µg/mL insulin, 6.25 µg/mL transferrin, 5.33 µg/mL linoleic acid, 1.25 µg/mL bovine serum albumin, 10^− 7^ M dexamethasone, 50 µg/mL ascorbate-2 phosphate, 1 µM sodium pyruvate, 100 µg/mL penicillin and 10 µg/mL streptomycin, in the presence of TGF-β3 (10 ng/mL). Pellet cultures were maintained at 37 °C, 5% CO_2_ and chondrogenic media were replaced every 3–4 days. Pellets of human chondrocytes were differentiated for 3 weeks. For histochemistry 3D pellets were fixed with 4% paraformaldehyde in PBS for 10 min and embedded in paraffin. Sections were deparaffinized, rehydrated, and glycosaminoglycan accumulation in cultured chondrocytes was assessed by Alcian Blue staining. HEK293 (wild type) and HEK293 stably expressing P2X7A (namely, HEK293-P2X7A) were grown in standard culture medium as above reported [31].

### Immunocytochemistry

Immunocytochemistry analysis was performed employing the ImmPRESS reagent kit (Vectorlabs, Burlingame, CA). Cells were fixed with cold 100% methanol for 10 min, permeabilized with 0.2% (v/v) Triton X-100, in TBS 1X (Tris-buffered saline) for 5 min and washed with TBS 1X. After blocking with 2% normal horse serum (Vectorlabs) for 10 min at room temperature (RT), the different primary antibodies were added and incubated at 4 °C overnight. Cells were then washed with TBS 1X and incubated in Vecstain ABC reagent (Vectorlabs) for 30 min and stained with DAB solution (Vectorlabs). After washing, cells were mounted in glycerol/TBS 9:1 and observed using a NikonEclipse 50i Brightfield microscope from Nikon Corporation (Tokyo, Japan). Quantitative image analysis of immunostained cells was obtained using a computerized video camera-based image analysis system (with ImageJ software, http://fiji.sc/Fiji) under brightfield microscopy. At least 10 fields per replicate were subjected to densitometric analysis. We performed the quantification of pixels per 100 cells and not per area in order to take into account the different cell morphologies and confluences [32].

### In vitro measure of ATP levels

Extracellular ATP (eATP) levels were quantified using the ENLITEN^®^ rLuciferase/Luciferin reagent (#FF2021, Promega, Milan, Italy). Chondrocytes were seeded at a density of 2 × 10^4^ cells per well in 24-well plates (#655077, Greiner Bio-One, Milan, Italy). After allowing cell attachment, the culture medium was removed and replaced with 100 µL of fresh serum-free medium, followed by incubation for 1 h at 37 °C. Subsequently, 100 µL of rLuciferase/Luciferin reagent was added to each well. Luminescence (RLU/s) was measured using a Tecan Spark multimode microplate reader (Tecan Group Ltd., Männedorf, Switzerland) and normalized to total cellular protein content (µg). 

### Immunogold labeling and electron microscopy

Chondrocytes (2 × 10^6^) were harvested by trypsinization. The cell suspension was fixed in 2% paraformaldehyde/PBS for 1 h, permeabilized with 0.1% Triton X-100 and blocked with PBS/2% bovine serum albumin (BSA) [33]. Cells were labelled overnight with anti-P2X7R (# P8232, Merck KgaA, rabbit anti-human, 1: 20 dilution). Samples were then incubated with Protein A-20 nm Colloidal Gold Labeled. Finally, cells were fixed in glutaraldehyde 2.5% phosphate buffer and osmium tetroxide 2%, dehydrated and araldite embedded. The ultra-thin sections of a selected area were contrasted with uranyl acetate lead citrate, and observed with a Zeiss EM910 transmission electron microscope (ZEISS, Jena, Germany). Images were captured using an Olympus Megaview III digital camera (Olympus Co., Tokyo, Japan).

### P2X7R transfection

Human chondrocytes were seeded according to the experimental application. For gene expression analyses, cells were plated in 12-well plates at a density of 4 × 10^4^ cells/well. For live-cell imaging experiments, cells 1 × 10^5^ cells/well were seeded onto sterilized 24 mm (Ø) glass coverslips. The following day, when cells reached approximately 60–70% confluence, transfection was performed using 400 ng/ml of the eGFP-hP2X7R expression vector and Lipofectamine 2000 (Thermo Fisher Scientific), according to the manufacturer’s instructions. At 24 h post-transfection, cells plated in 12-well plates were harvested for RNA extraction and analysis by RT-qPCR. For imaging experiments, live-cell imaging was performed 24 h post-transfection under controlled environmental conditions (37 °C, 5% CO₂) to confirm expression of the eGFP-hP2X7R construct. During acquisition, cells were maintained in modified Krebs–Ringer buffer (mKRB) containing 135 mM NaCl, 5 mM KCl, 0.4 mM KH₂PO₄, 1 mM MgSO₄, 20 mM HEPES, 5.5 mM glucose, and 1 mM CaCl₂ (pH 7.4). Nuclei were stained with Hoechst 33258 (2 µg/mL, #94403, Merck). Images were acquired using an FV3000 confocal laser scanning microscope (Evident Europe GmbH, Germany) equipped with a PlanAPON 60×/NA 1.4 oil-immersion objective. eGFP was excited at 488 nm, and images were collected with a pixel size of 207.2 nm (104 nm with zoom).

### Subcellular fractionation

For subcellular fractionation, cell pellets were resuspended in Buffer A (0.1 M Tris/MOPS, 0.1 M EGTA/Tris, 1 M sucrose) supplemented with a protease inhibitor cocktail (Merck KgaA). Cells were homogenized by passing them 15 times through a 25-gauge needle, then centrifuged at 8,000 g for 10 min at 4 °C. The resulting supernatant was collected as the cytoplasmic fraction. The nuclear pellet was resuspended in Buffer B (20 mM HEPES, pH 7.9, 25% glycerol, 0.42 M NaCl, 1.5 mM MgCl_2_, 0.2 mM ethylenediaminetetraacetic acid (EDTA), 0.5 mM dithiothreitol, and 0.5 mM phenylmethylsulfonyl fluoride), also supplemented with protease inhibitors. After 30 min of incubation on ice, nuclear extracts were obtained by centrifugation at 12,000 rpm for 5 min at 4 °C. Extracts were quantified using the Bradford protein assay (Bio-Rad Laboratories, CA, USA), then snap-frozen and stored at -80 °C for later analysis by Western blot.

### Coimmunoprecipitation (Co-IP) assay

The Co-IP assay was conducted as previously reported [34]. Briefly, chondrocytes were lysed in ice-cold (RIPA) buffer [50 mMTris-HCl, pH 7.8, 1%NP-40, 150 mM NaCl, 0.5% SDC (sodium deoxycholate), and 0.1% SDS (sodium dodecyl sulfate)] supplemented with protease inhibitors cocktail for 15 min at 4 °C. The cells were centrifuged at 12,000 g for 10 min at 4 °C to remove cell debris. The Bradford method was used for protein quantification. 5% of the cell lysates were kept as the input sample, and 1 mg of protein lysates was incubated with 10 µg of the specific primary antibodies overnight at 4 °C, followed by incubation with 20 µL of Magna ChIP Protein A + G magnetic beads for 2 h at 4 °C with gentle rocking. Normal rabbit IgG (#2729S, Cell Signaling Technology, Danvers, MA, USA) was used as negative control. The beads were collected by centrifugation at 6,000 rpm for 1 min at 4 °C and washed four times with RIPA buffer and once with PBS 1X. The immunocomplexes were released by heating the beads in 15 µL of Laemmli sample buffer and subjected to Western blot analysis.

### Western blot analysis

Protein separation was performed on 7% SDS-polyacrylamide gels. The separated proteins were then transferred onto a Immobilon-P PVDF membrane (Merck KGaA). The membranes were blocked for 1 h with a 5% non-fat milk in Tris buffered saline containing 0.1% Tween-20 0.1% (TBST) to minimize nonspecific binding. After that, the membranes were incubated with primary antibodies (see Supplementary Table 1) in 5% non-fat milk overnight at 4 °C on a shaking platform. Afterward, the membranes underwent 3 × 10 min washes with TBST and were subsequently exposed to specific secondary antibodies diluted in non-fat milk 0.1% TBST for 1 h at RT. Membranes were washed three times with TBST and then incubated for 1 h at RT with the appropriate HRP-conjugated secondary antibodies: goat anti-rabbit, # sc-2030, 1:20000; goat anti mouse, # sc-2005, all from Santa Cruz Biotechnology. The blots were developed using enhanced chemiluminescent solution (Merck KGaA) and visualized with chemiluminescent Western blot imaging systems (ChemiDoc MP imaging system, Biorad, California, USA).

### Immunofluorescence imaging

Cells (2 × 10^4^/well) were seeded on sterilized glass coverslips. After 24 h, cells were washed once with PBS and fixed with 4% paraformaldehyde for 10 min at 37 °C. For permeabilization, the cells were treated with PBS-0.05% Triton™ X-100 at RT for 10 min. For blocking, the cells were incubated with 4% BSA at RT for 30 min. Then, cells were incubated overnight with primary antibodies (see Supplementary Table 1) at 4°C. The next day, after washing three times with PBS, the cells were incubated with secondary antibodies (Alexa Fluor 488 goat anti-rabbit and Alexa Fluor 546 goat anti-mouse (1:1000; Thermo Fisher Scientific) for 1 h at RT. Finally, the coverslips were washed 3 times with PBS and mounted with ProLong Gold Antifade with DAPI (4′,6-diamidino-2-phenylindole) (# P36935, Thermo Fisher Scientific). Samples were acquired using a FV3000 confocal laser scanning microscope (Evident Europe GmbH) equipped with a PlanAPON60X/N.A.1.4 oil-immersion objective. Fluorophores were excited using 488 and 561 nm laser lines, and pixel size was set at 207.2 nm. Colocalization analysis was performed using the JaCoP plugin in ImageJ and the Pearson correlation coefficient was calculated.

### Proximity ligation assay (PLA)

Proximity ligation assays (PLA) were performed using the Duolink^®^ In Situ Red Kit Mouse/Rabbit (# DUO92102, Merck KGaA). Briefly, cells were seeded on 13 mm (Ø) glass coverslips for 24 h and fixed with 4% paraformaldehyde (PFA) at RT for 10 min. Subsequently, cells were washed four times with PBS for 5 min, permeabilized with 0.05% Triton X-100 in PBS for 10 min. Next, the cells were blocked with 4% BSA in PBS for 30 min at RT and incubated with the primary antibodies at RT for 1 h in a humidified chamber. Then, cells were washed twice with PBS for 5 min and incubated with pre-diluted Minus and Plus PLA probes/complementary oligonucleotides in Antibody Diluent at 37 °C for 1 h. Cells were washed twice with Wash Buffer A for 5 min and then incubated with ligation solution at 37 °C for 30 min. After ligation, samples were again washed twice with Wash Buffer A for 5 min at RT and incubated with amplification-polymerase solution at 37 °C for 100 min. Finally, cells were washed twice with Wash Buffer B at RT for 10 min, and briefly with Wash Buffer B 0.01X, subsequently coverslips were mounted on the slide. Nuclei were counterstained with DAPI (1 µg/ml; # MBD0015, Merck KGaA). Samples were acquired using a FV3000 confocal laser scanning microscope (Evident Europe GmbH) equipped with a PlanAPON60X/N.A.1.4 oil-immersion objective. Fluorophores were excited using 405 and 561 nm laser lines, and pixel size was set at 207.2 nm. The number of PLA puncta per nucleus was quantified using ImageJ’s Analyze Particles tool and expressed as median. Details of all the different antibodies and dilutions used in PLA assays are indicated in the Supplementary Table 1.

### RNA isolation and RT-qPCR

Total RNA was isolated from chondrocytes using the RNeasy micro kit (# 74004, Qiagen GmbH, Hilden, Germany) according to the manufacturer’s instructions. A NanoDrop™ ND1000 UV-VIS spectrophotometer (Isogen Life Science B.V., Utrecht, Netherlands) was used to measure RNA quantity. cDNA was synthesized from total RNA in a 20 µL reaction volume using the High-Capacity cDNA RT kit (Thermo Fisher Scientific), according to the manufacturer’s instructions. qPCR analysis was performed using both TaqMan probe assay (Thermo Fisher Scientific) and SYBR Green chemistry using gene-specific primers, depending on the target gene. Experimental reactions were conducted on a CFX96™ PCR detection system (Bio-Rad Laboratories, Inc., CA, USA), in a final volume of 10 µL. For TaqMan assays, PCR reactions contained universal master mix II, the specific probe, and cDNA template. For SYBR Green assays, reactions included iTaq Universal SYBR Green Supermix (Bio-Rad), forward and reverse primers, and cDNA template. Thermal cycling conditions consisted of an initial denaturation step at 95 °C for 10 min followed by 40 cycles of denaturation at 95 °C and annealing/extension at 60 °C. For SYBR Green assays, a melt curve analysis was performed at the end of each run to confirm amplification specificity. Gene expression levels were normalized to the reference gene GAPDH, and relative expression was calculated using the 2^⁻ΔΔCt^ method. All reactions were performed in technical duplicates, with three independent biological replicates analyzed.

### Chromatin Immunoprecipitation (ChIP)

ChIP assays were performed as previously described [34]. Briefly, 5 × 10^7^ chondrocytes cultured under 2D or 3D conditions were fixed for 10 min at 37 °C in culture medium supplemented with 1% formaldehyde. Cells were then rinsed three times with ice-cold PBS and lysed on ice in SDS lysis buffer (50 mM Tris-HCl (pH 8.0), 10 mM EDTA, 1% SDS, supplemented with 1X protease inhibitor cocktail (Merck KgaA). Chondrocytes cultured in 3D cell pellets formed large cellular aggregates. For ChIP analysis, the pellets were homogenized in SDS lysis buffer using a small disposable plastic pestle and an aliquot of Molecular Grinding Resin (G-Biosciences, St. Louis, MO, USA). The isolated chromatin was sonicated to an average fragment size of approximately 200–1000 bp, (12 cycle, 10s pulses; 40% amplitude) using a “VibraCell” VC 130 (Sonics, Newtown, CT, United States). Sonicated chromatin was cleared by centrifugation at 13,000 rpm for 10 min and then diluted 10-fold with dilution buffer (20 mM Tris-HCl pH 8.0, 1.2 mM EDTA, 0.01% SDS, 1.1% Triton X100, 167 mM NaCl, and protease inhibitor mixture). Protein/DNA complexes were incubated on a rotating platform overnight at 4 °C using 5 µg of primary antibodies against P2X7R and lamin A/C and 20 µL of Magna ChIP Protein A + G magnetic beads. Immunoprecipitated chromatin complexes were sequentially washed with the following buffers: low salt wash buffer (0.1% SDS, 1% Triton X-100, 2 mM EDTA, 20 mM Tris–HCl pH 8.1,150 mM NaCl), high salt wash buffer (0.1% SDS, 1%Triton X‐100, 2 mM EDTA, 20 mM Tris–HCl pH‐8.1, 500 mM NaCl), LiCl wash buffer (0.25 mM LiCl, 1% IGEPAL‐CA630, 1% deoxycholic acid, 1 mM EDTA,10 mM Tris‐pH 8.1), and TE buffer. All wash procedures were performed with a magnetic separator. Immunocomplexes were eluted by adding 200 µL of a freshly prepared solution containing 1% SDS in 0.1 M NaHCO_3_, followed by incubation at RT for 30 min. Cross-linking was reversed by adjusting the final NaCl concentration to 0.2 M, followed by incubation at 65 °C for 6 h. Samples were subsequently treated with proteinase K for 1 h at 45 °C, and DNA was purified using the Wizard^®^ SV Gel and PCR Clean-Up System kit (Promega, #A9281, Madison, WI, USA), according to the manufacturer’s instructions. qPCR was performed with CFX96 real-time detection system (Bio-Rad) using iTaq Universal SYBR Green SuperMix (Bio-Rad). The Input fractions (1% of the cell lysate before immunoprecipitation) were used as the internal positive control. Specific primers for different regions of gene promoters were designed or obtained from previous reports [35] (see Supplementary Table 2). Each promoter region was analysed in duplicate qPCR reactions per experiment, and three independent ChIP experiments were performed. ChIP-qPCR data were calculated as a fold enrichment using the 2^−ΔΔCt^ method and normalized to the IgG signal; less than 3-fold enrichment was considered as weak or no binding.

### Primer design

Primers were designed by the Primer3Plus web client (https://www.primer3plus.com/). Amplicon and primer size was limited to 60–150 and 18–24 base pairs, respectively. Melting temperature was set between 60 and 63 °C and GC-content was limited to 40–60%. Primers were checked for possible hairpin and primer dimer structures in Net Primer web (https://www.premierbiosoft.com/netprimer/). Primers were subsequently purchased from Integrated DNA Technologies (Coralville, USA).

### Statistical analysis

All experiments were performed with at least three biological replicates. Differences between two groups were evaluated using the non-parametric Mann-Whitney U test. Data are presented as mean ± standard deviation (SD). Graphical representations and statistical analyses were performed using GraphPad Prism (version 8.0). Significance thresholds were set at *P < 0.05.

## Results

### Increased P2X7R expression is associated with expansion of chondrocytes in culture

**Fig. 1:**
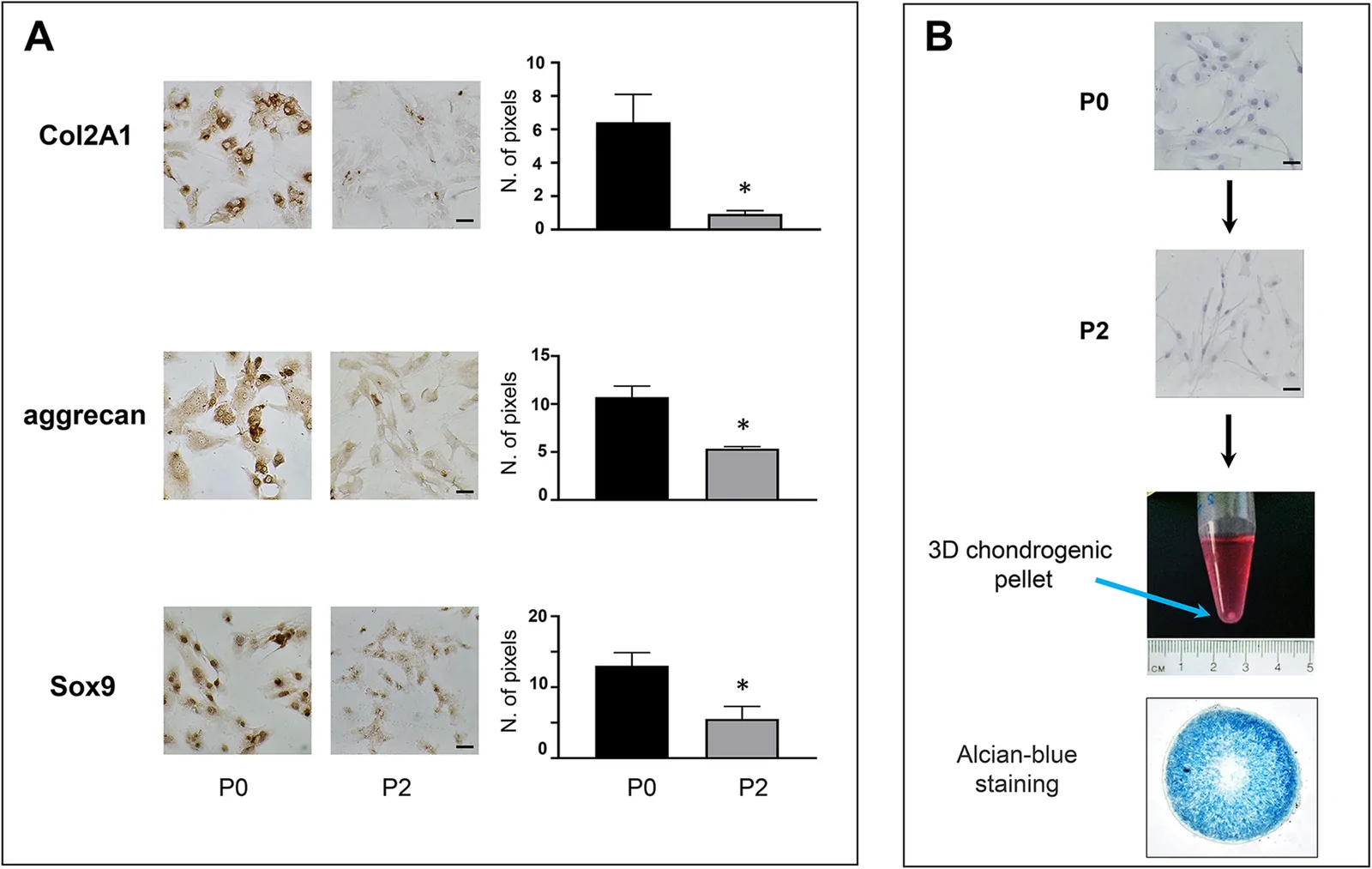
Characterization of human freshly isolated P0 and expanded P2 chondrocytes. **A** The expression of cartilage-specific markers (Col2A1, aggrecan, and Sox9) was assessed by immunocytochemistry in chondrocytes during the dedifferentiation process, from passage 0 (P0) to passage 2 (P2). Representative optical photomicrographs are reported. Protein levels were quantified by densitometric analysis of immunocytochemical pictures using ImageJ software and expressed as means of pixels per one hundred cells. Data are presented as the mean ± SD (*n* = 3). *P < 0.05 vs. P0. *Scale bar = 20 μm*. **B** Cell morphology of P0 and P2 chondrocytes was evaluated by hematoxylin staining. Representative optical photomicrographs are shown. *Scale bar = 20 μm*. Proteoglycan content was evaluated by Alcian Blue staining performed on 3D pellet cultures of P2 chondrocytes maintained in chondrogenic medium for 21 days

In order to obtain an adequate number of cells, P0 chondrocytes freshly isolated from different donors [36] were expanded as described in the Materials and methods section, and characterized as reported in Fig. 1. Compared to freshly isolated P0 chondrocytes, expanded P2 chondrocytes tend to express lower levels of chondrogenic markers, including type II collagen (Col2A1), aggrecan, and the master regulator of chondrogenesis Sox9 (Fig. 1A). As expected, the shape of the cells monitored during the culture passages tends to change from round to flattened (Fig. 1B). When cultured in a three-dimensional (3D) culture system, such as chondrogenic pellet, expanded P2 chondrocytes are able to aggregate and produce Alcian blue-positive extracellular matrix (ECM), indicating the presence of sulfated glycosaminoglycans (GAGs), a key component of cartilage ECM (Fig. 1B). This is the proof that these cells, even after expansion in monolayer culture, retain the potential to re-differentiate and form cartilage-like tissue in a 3D condition.

**Fig. 2:**
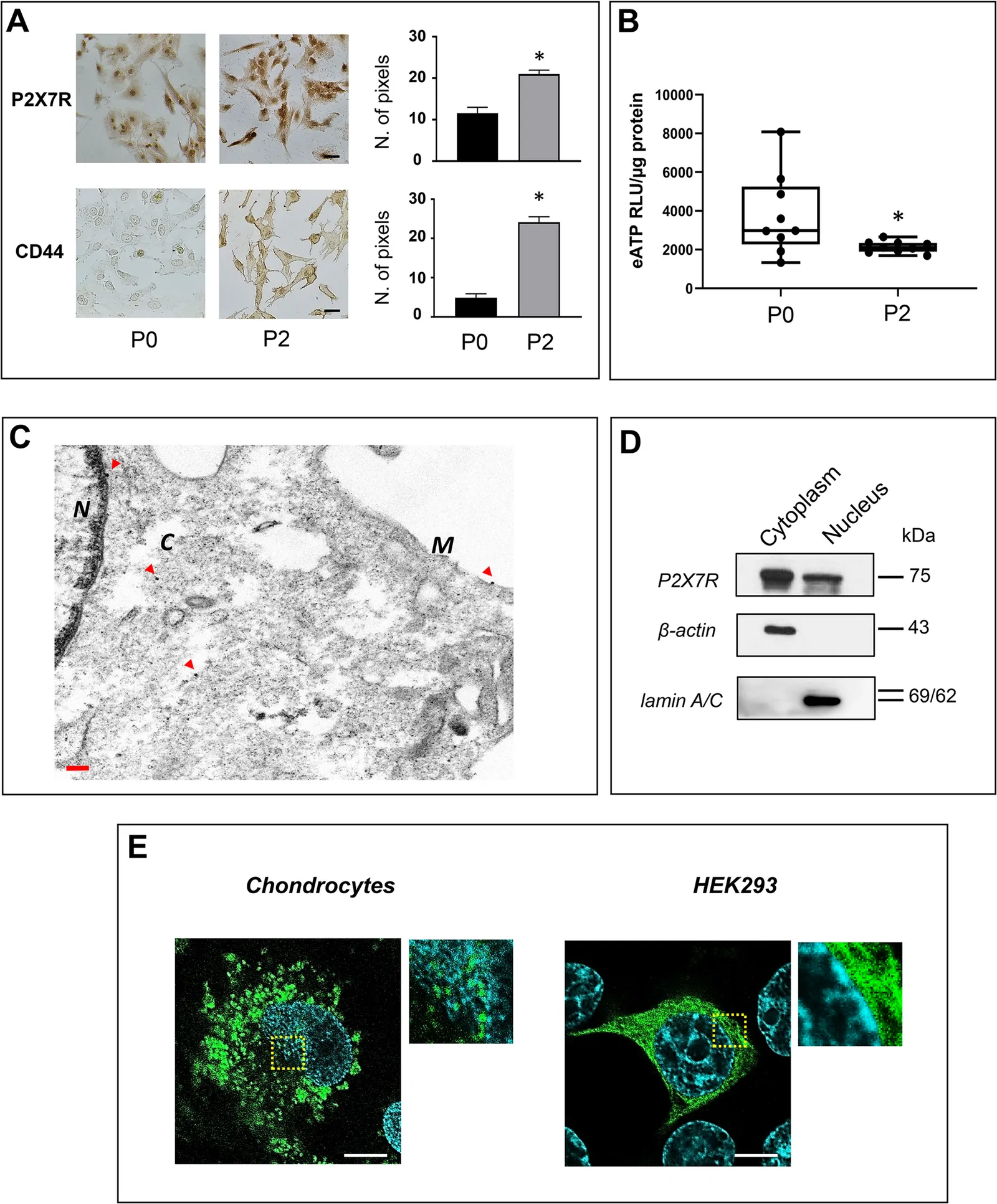
Expression and subcellular localization of P2X7R in human chondrocytes. **A** The expression of P2X7R and its positive allosteric effector, CD44, was assessed by immunocytochemistry in chondrocytes during the dedifferentiation process, from passage 0 (P0) to passage 2 (P2). Representative photomicrographs are reported. Protein levels were quantified by densitometric analysis of immunocytochemical images using ImageJ software and expressed as means of pixels per one hundred cells(mean ± SD; *n* = 3). *P < 0.05 vs. P0. *Scale bar = 20 μm*. **B** Extracellular ATP (eATP) levels were measured in chondrocytes at passages P0 and P2 using a bioluminescent assay and are presented as relative light units (RLU) per µg protein. Data are reported as Whisker box plot representing min to max (line indicates median, *n* = 3 with three technical replicates). *P < 0.05 vs. P0. **C** The presence of P2X7R was revealed in the nucleus (N), cytoplasm (C) and cellular membrane (M) by immunogold staining and TEM observation in chondrocytes at passage 2. A representative image (*n* = 3) is shown with the gold particles indicated by the red arrowheads. *Scale bar = 100 nm*. **D** The presence of P2X7R was confirmed by Western blot analysis of cytoplasmic and nuclear extracts of chondrocytes at passage 2. β-actin and lamin A/C were used as markers for the cytoplasmic and nuclear fractions, respectively (*n* = 3). **(E)** Chondrocytes at passage 2 and HEK293 cells were transiently transfected with 400 ng/ml of the eGFP-hP2X7R construct. Green fluorescence indicates the expression and subcellular localization of P2X7R. Blue fluorescence indicates nuclei stained with Hoechst 33258. Representative images (*n* = 3) together with high-magnification pictures are shown. *Scale bar = 10 μm*

As previously demonstrated, the P2X7R is present in chondrocytes [10, 19] and can be activated by extracellular ATP (eATP) [20, 21]. Here we found that P2X7R expression increased during cell expansion (Fig. 2A), likely due to its potential involvement in stimulating cell proliferation and the dedifferentiation process [15, 20]. Measurement of eATP showed that P0 chondrocytes released appreciable levels of this signaling molecule, which is indeed the primary ligand for P2X7R and, even under basal conditions, can influence a wide range of cellular processes, including cellular metabolism and matrix turnover (Fig. 2B) [37]. However, eATP levels decreased significantly during cell expansion (Fig. 2B). How P2X7R relates to these low eATP levels can be explained by several factors. The reduced eATP levels in P2 chondrocytes could be compatible with the involvement of P2X7R in ATP-independent signaling pathways [38]. Alternatively, it is possible that P2X7R sensitivity to eATP variations is influenced by changes in the microenvironment, where, for example, the presence of particular effectors could play a critical role in terms of affinity [7, 38, 39]. This hypothesis is indirectly supported by our observation regarding CD44, a positive allosteric effector of P2X7R [40]. As reported in Fig. 2A, CD44 expression, almost undetectable in P0 chondrocytes, increased significantly in P2 chondrocytes. Taken together, this could explain how context can in principle influence the dual role of the P2X7R, which can both support cartilage degradation associated with loss of chondrocyte differentiation and maintain homeostasis and cell-to-cell communication in normal chondrocytes, employing different mechanisms of action yet to be fully understood.

### Subcellular distribution of P2X7R in chondrocytes

Considering how cellular localization influences protein function, especially in light of recent evidence on the pleiotropic effect of purinergic receptors, the presence of P2X7R in chondrocytes was further investigated by analyzing its subcellular distribution through different experimental approaches. Immunogold and TEM observation showed the presence of P2X7R in the cytoplasm and nucleus of chondrocytes, in addition to its canonical localization on the plasma membrane (Fig. 2C). This was validated by Western blot analysis of cytoplasmic and nuclear extracts, which demonstrated the presence of the full-length 68.9 kDa P2X7R in both compartments (Fig. 2D). To validate the fractionation process, β-actin and lamin A/C were used as loading controls for the cytoplasmic and nuclear fractions, respectively. The subcellular distribution of P2X7R was maintained even in P2 chondrocytes overexpressing the receptor after transfection with the construct encoding the full-length P2X7R (Fig. 2E). To exclude that the presence in the nucleus is due, for some reasons, to high expression levels, we performed the same analysis on P2X7R-negative human embryonic kidney HEK-293 cells. As shown in Fig. 2E, P2X7R-transfected HEK-293 cells, showed high levels of P2X7R expression both on the cell membrane and within the cytoplasm, but not in the nucleus. This suggests that nuclear positivity should be considered typical only of certain cells, such as chondrocytes.

### P2X7R interacts with nuclear proteins

**Fig. 3:**
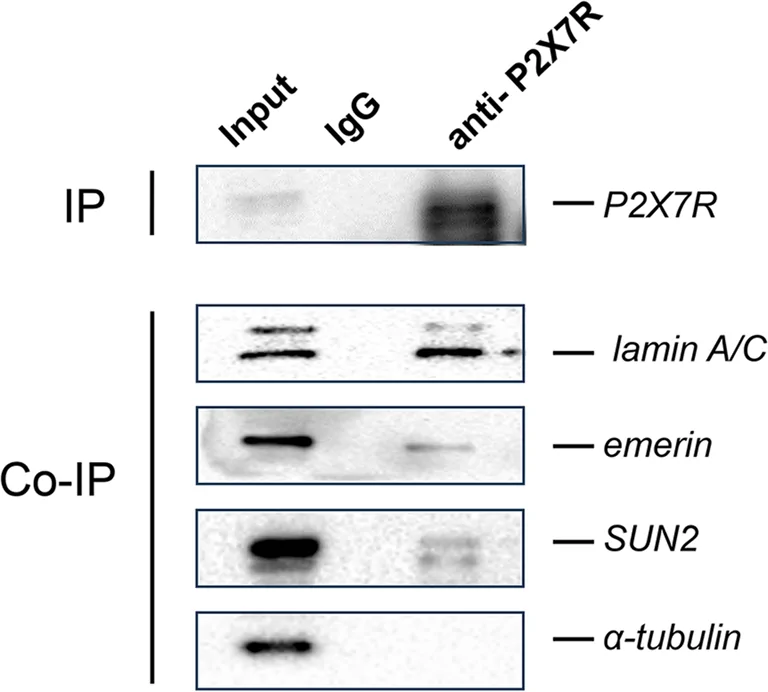
Association between P2X7R and specific nuclear proteins. Whole-cell lysates from chondrocytes at passage 2 were immunoprecipitated with an anti-P2X7R antibody, followed by immunoblotting with antibodies against lamin A/C, emerin, SUN2, and α-tubulin. 5% of the whole cell extract used for immunoprecipitation was loaded as input control. Lysates immunoprecipitated with an anti-IgG antibody were used as a negative control

Subsequent experiments were planned to understand the significance of the presence of P2X7R in the nucleus of the cells through co-immunoprecipitation (Co-IP) and proximity ligation (PLA) assays. In recent years, experimental approaches aimed at identifying partners with which the P2X7R can interact have led to the formulation of various hypotheses regarding the molecular mechanisms underlying its pleiotropic effects in various target cells [7, 41]. In the study described here, too, the goal was to identify potential partners of the receptor that could explain its possible, yet undescribed, involvement in nuclear phenomena. The study focused on three nuclear proteins - lamin A/C, emerin and SUN2 - on which there is extensive literature. Lamin A/C, located both in the nuclear envelope and within the nucleus, emerin, present predominantly in the inner nuclear membrane, and SUN2, located on the inner nuclear membrane, are all key proteins in organizing chromatin within the nucleus, controlling gene expression, and maintaining proper nuclear structure and function [25–28]. Using specific antibodies and developing appropriate protocols for primary cells in culture, we demonstrated an association between each of these proteins and P2X7R. Co-immunoprecipitation experiments revealed a clear interaction between lamin A/C and P2X7R, emerin and P2X7R, and SUN2 and P2X7R (Fig. 3).

**Fig. 4:**
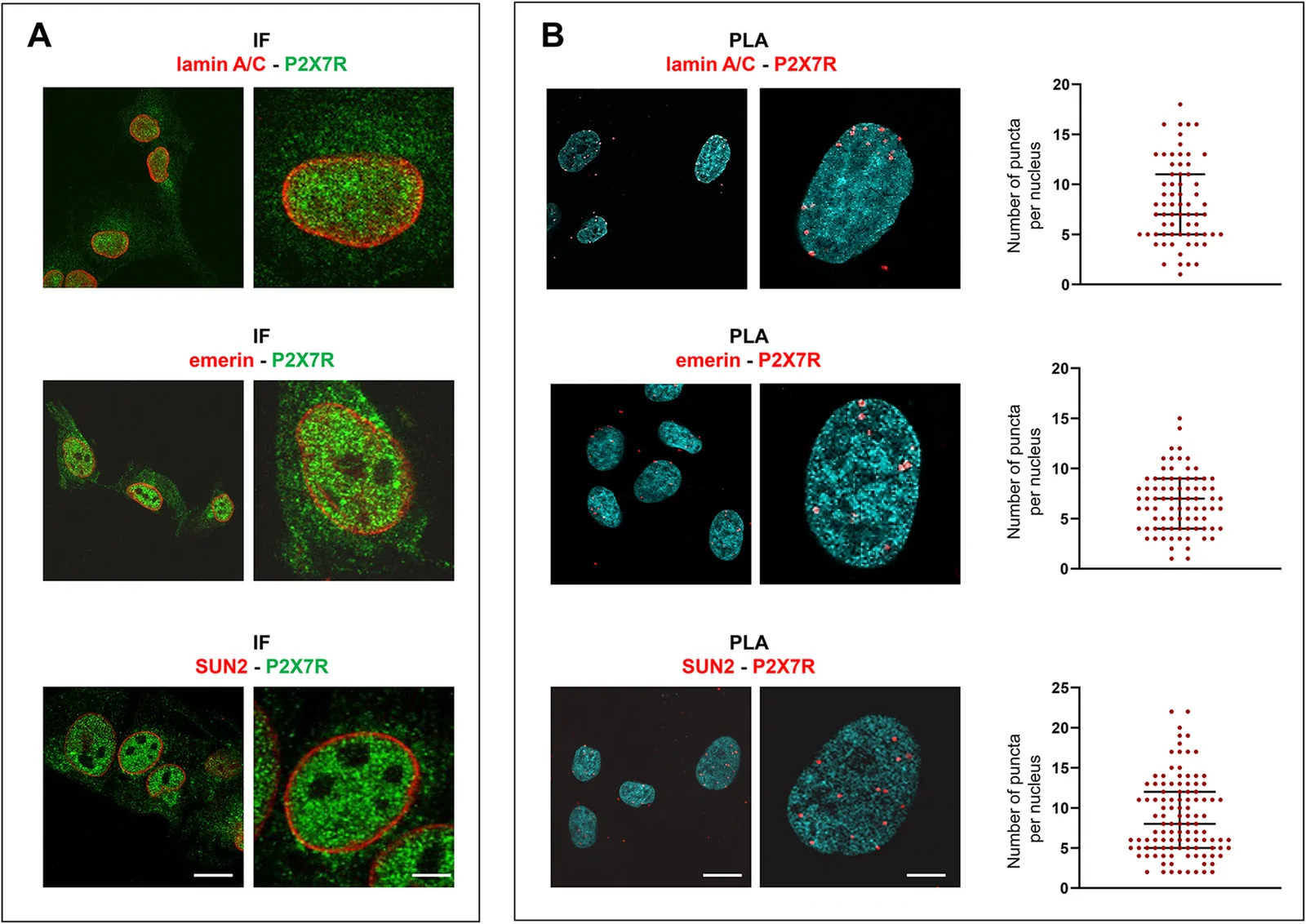
Colocalization between P2X7R and specific nuclear proteins. **A** The chondrocytes were co-labeled with anti-P2X7R antibody (green) and anti-lamin A/C, anti-emerin, and anti-SUN2 antibody (red). Representative images of immunofluorescence detection (*scale bar = 10 μm*) together with high-magnification pictures (*scale bar = 2 μm*) are reported. Merge images represent an overlay of the two channels where colocalization is indicated by a color change (yellow). **B** The close proximity of P2X7R to lamin A/C, emerin and SUN2 was determined by PLA. Representative merged images (*scale bar = 10 μm*) together with high-magnification pictures (*scale bar = 2 μm*) are reported. Protein interactions are revealed as red dots, each representing a single protein-protein interaction event. Nuclei were counterstained with DAPI (blue). Quantification of PLA signals is expressed as the number of puncta per nucleus, and the horizontal bars indicate the median with interquartile range (*n* = 3)

These associations between P2X7R and the proteins described above was then validated investigating subcellular localization with PLA, a technique that allows us to highlight even transient events and low-affinity interactions of endogenously expressed proteins. A first series of immunofluorescence colocalization experiments showed a partial colocalization between P2X7R and lamin A/C (Pearson’s coefficient 0.41), P2X7R and emerin (Pearson’s coefficient 0.24), and P2X7R and SUN2 (Pearson’s coefficient 0.32), as reported in Fig. 4A. These values are consistent with the broad subcellular distribution of P2X7R, indicating partial colocalization with nuclear envelope markers.

Accordingly, protein interactions within their natural cellular context, investigated through PLA, provided us with a series of images indicating that the P2X7R and lamin A/C, emerin and SUN2 are in close proximity in both the nuclear membrane and in the nucleoplasm (as revealed by PLA puncta reported in Fig. 4B). The quantification of PLA puncta per nucleus showed that the interactions examined are not significantly different from each other: with lamin A/C and emerin identical median values were observed (median = 7), while with SUN2, P2X7R appeared to be associated slightly more often (median = 8). A negligible signal was detected in control experiments in which the anti-tubulin antibody was used in combination with the anti-P2X7R antibody (Supplementary Figure S1).

### P2X7R is recruited at specific gene promoters

**Fig. 5:**
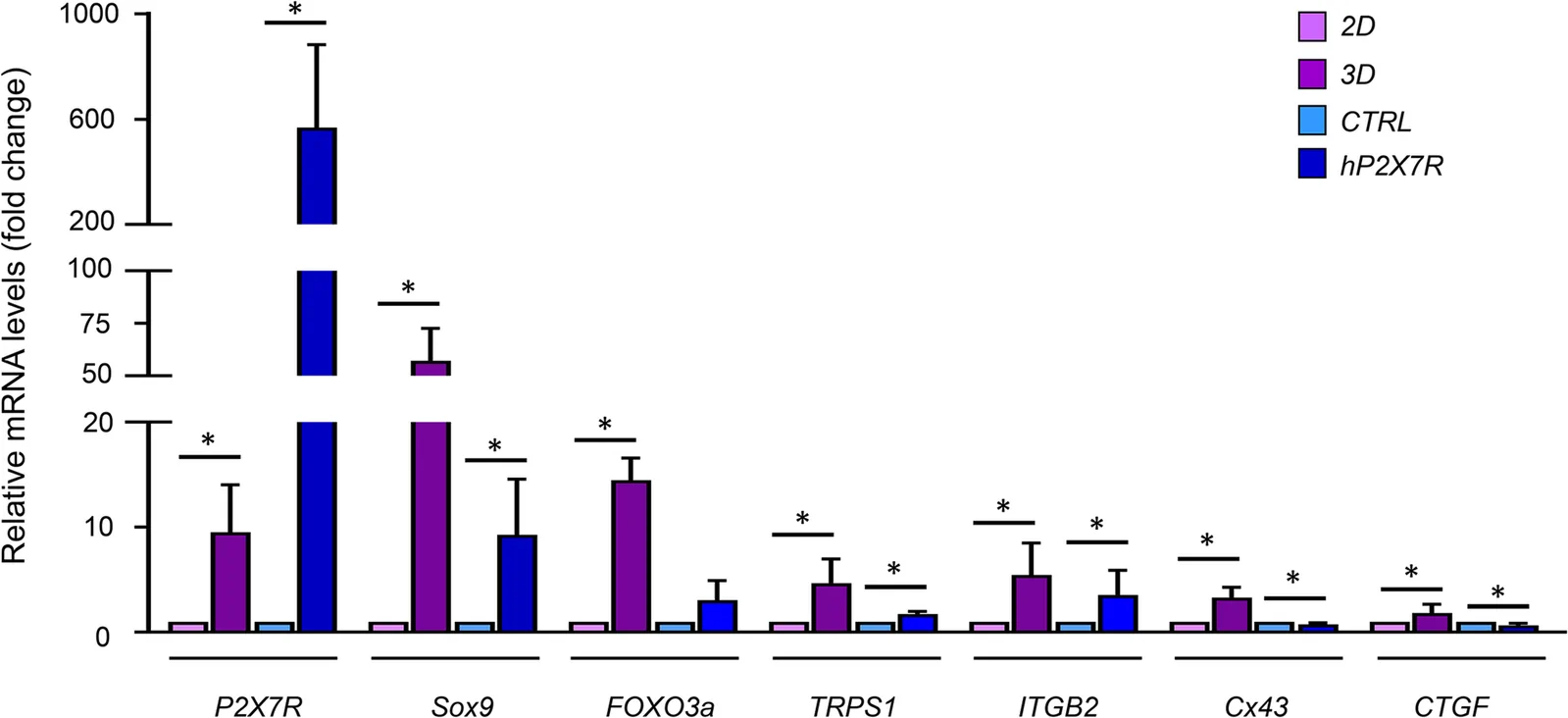
Effects of P2X7R overexpression and 3D culture conditions on gene expression in human chondrocytes. Chondrocytes at passage 2 were cultured under 2D and 3D conditions or transiently transfected with a human P2X7R (hP2X7R) expression construct. Relative mRNA expression levels of P2X7R, Sox9, FOXO3a, TRPS1, ITGB2, Cx43, and CTGF were quantified by real-time RT-qPCR. For each gene analyzed, expression in 3D culture is presented as fold change relative to 2D condition, whereas expression in hP2X7R-transfected cells is presented as fold change relative to untransfected control cells (CTRL). Gene expression levels were normalized to GAPDH as an internal control. Data are shown as mean ± SD (*n* = 3). Statistical significance is indicated as *P < 0.05 (2D vs. 3D; CTRL vs. hP2X7R)

**Fig. 6:**
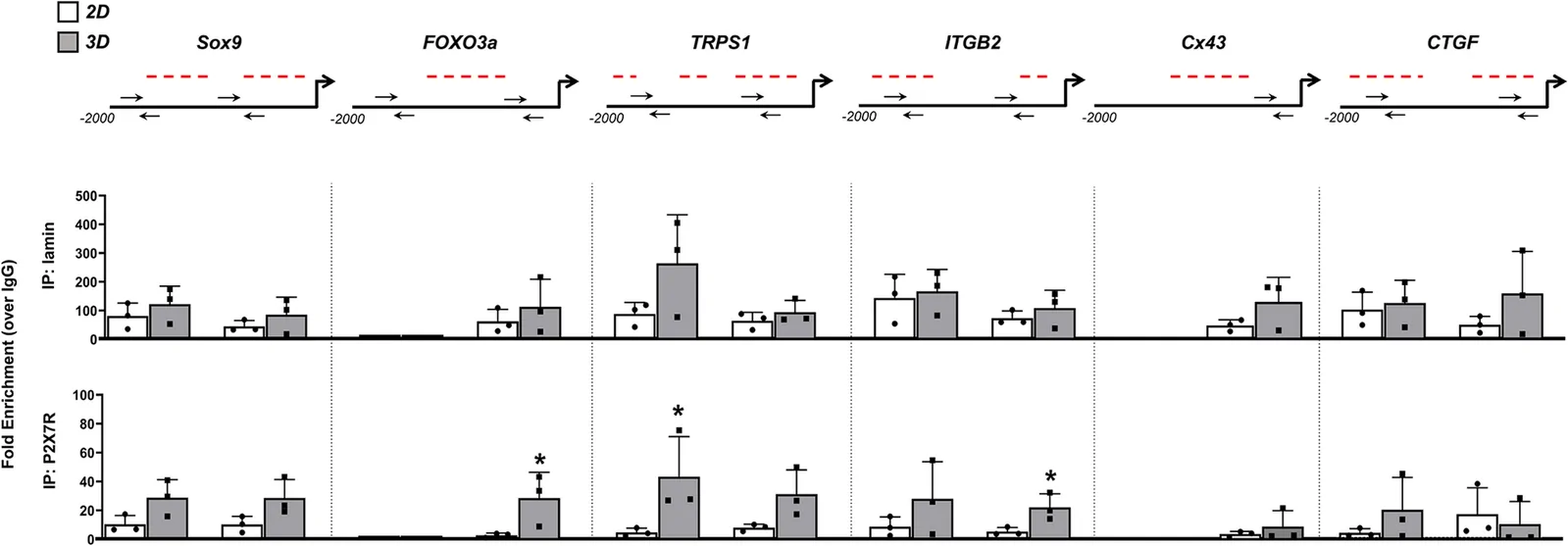
Recruitment of P2X7R at specific gene promoters. Chromatin immunoprecipitation followed by qPCR was performed to assess lamin A/C and P2X7R occupancy at the regulatory region located within 2000 base pairs upstream of the + 1 transcription start site of Sox9, FOXO3a, TRPS1, ITGB2, Cx43, and CTGF genes, in chondrocytes grown in 2D and 3D culture conditions. The potential binding regions of lamin A/C are shown above each promoter at the top of the figure (red dashed line). The positions of specific primers used for qPCR amplification are also reported. ChIP-qPCR data were analyzed using the 2^−ΔΔCT^ method, normalized to input DNA, and expressed as fold enrichment relative to the IgG negative control. Data are presented as mean ± SD (*n* = 3). *P < 0.05 vs. 2D. Bars represent mean values, with individual data points included

The association between P2X7R and nuclear proteins we found raise new and intriguing questions about the novel and unexpected roles of P2X7R, which deserve in-depth investigation combining various biochemical and functional approaches. The preliminary experiments described below were dedicated to explore the potential involvement of P2X7R in the transcriptional control program through ChIP experiments, probing regulatory zones of specific genes. We focused on a group of genes responsible for chondrogenic differentiation, such as: (i) three transcription factors including Sox9, the master regulator of chondrogenesis [42]; FOXO3a, one of the best-known genes associated with longevity and protection against chondrocyte dysfunction [43]; TRPS1, a regulator of chondrocyte proliferation, maturation, and apoptosis [44]; and (ii) three mediators of ECM function including integrin beta-2 (ITGB2), a transmembrane receptor involved in cell adhesion and cartilage development and maintenance [45]; connexin 43 (Cx43), an integral membrane protein that forms hemichannels essential for the proper function and maintenance of mature cartilage [46]; connective tissue growth factor (CTGF/CCN2), a matricellular-secreted protein required for ECM production [47]. As shown in Fig. 5, the transcription of all these genes along with that of P2X7R was positively affected by the chondrogenic 3D culture pellet, a form of scaffold-free 3D culture, which is commonly used to stabilize the chondrogenic potential of chondrocytes cultured in vitro [48]. This leads to the induction and consolidation of a gene expression profile similar to that found in vivo through the concerted action of multiple factors, making this condition ideal for analyzing gene regulatory regions through ChIP. To understand the impact of P2X7R on modulating the expression of these genes, the same analysis was performed on chondrocytes overexpressing the receptor after transfection with the construct encoding the full-length P2X7R. Interestingly, we found that the expression of Sox9, TRPS1, and ITGB2 genes was significantly increased, while that of FOXO3a increased but not significantly, and that of Cx43 and CTGF gene was significantly decreased. Therefore, the different effect that P2X7R overexpression has on the expression levels of the genes examined suggests that P2X7R may participate in their regulation in different ways. Based on these findings, we then moved on to ChIP experiments to pursue the above-mentioned objective, i.e. to demonstrate the possible participation of P2X7R in genomic events modulating chromatin organization and transcriptional control. For each gene, a promoter region of approximately 2000 nt upstream of the transcription start site (TSS) was considered. As expected, bioinformatic analysis revealed that each promoter contains numerous sequences associated with lamin A/C [49, 50], the protein with which this investigation began, being the best-characterized among nuclear proteins and capable of directly binding to chromatin [25, 30]. As shown in Fig. 6, ChIP assay indeed revealed the recruitment of lamin A/C within each promoter (Fig. 6). Interestingly, the presence of P2X7R was detected in these same regions: these signals are lower than those of the lamina A/C, but significant and consistently observed in all replicates and distinguishable from the background. Compared to 2D culture conditions, 3D culture condition tend to favor the recruitment of both lamin A/C and P2X7R. In particular, the difference was significant for the recruitment of P2X7R to the promoter of the FOXO3a, TRPS1, and ITGB2 genes.

## Discussion

P2X7R is one of the several plasma membrane proteins that include channels, transporters, enzymes, receptors, and anchors for cytoskeletal and ECM proteins in the articular chondrocytes. All these membrane proteins are crucial not only for the responsiveness of chondrocytes to signals from the microenvironment, but also for the development of receptors-based therapeutic approaches for the treatment of joint diseases, as some of them represent valuable drug targets. P2X7R, like other purinergic P2 receptor subtypes, is thought to participate in the mechanisms by which cells sense and respond to mechanical forces, through the binding of extracellular ATP (eATP) which acts as a signal to activate the receptor and initiate a response [51, 52]. It is known that the response of articular chondrocytes to mechanical load plays a fundamental role in both physiological and pathological conditions [15, 16, 18, 24]. On the one hand, physiological loading is crucial for joint health and cartilage integrity, as it acts through low eATP levels that have beneficial effects on chondrocyte growth/survival. Conversely, insufficient or excessive physical force combined with increased eATP can induce harmful processes such as inflammation, extracellular matrix degradation, and chondrocyte death. It should be noted that to date, most research regarding the role of P2X7R in cartilage concerns its involvement in inflammation and joint pain and proposes it as a potential therapeutic target for osteoarthritis and rheumatoid arthritis [19–21, 41, 53]. Little is known about how this receptor may participate in maintaining cartilage homeostasis. A contribution could come from studying the protein partners with which it interacts, the signaling pathways it can intercept, or from deciphering the meaning of its subcellular localization. It has been recently demonstrated that chondrogenic differentiation from Bone Marrow Mesenchymal Stromal Cells (BMMSCs) occurs through the activation of P2X7R-mediated phosphoinositide-3-kinase (PI3K)-AKT pathway [54], and that chondrocyte anabolism and homeostasis maintenance is supported by the P2X7R-mediated SIRT3 pathways [54]. This evidence strengthens the hypothesis that P2X7R might function as an energy receptor, also participating in early cartilage regeneration. This is consistent with previous studies defining P2X7R a key modulator of mitochondrial energy metabolism, demonstrating that it localizes to the mitochondria in several cell types and that its lack impairs oxidative phosphorylation (OxPhos) [13]. These data, combined with recent evidence describing the presence of P2X7R in various subcellular compartments, including the nucleus, adds further complexity to the intracellular physiology of P2X7R and prompted us to further investigate the potential unexplored roles of this receptor. After confirming the presence of P2X7R in the nucleus of chondrocytes, we sought to understand its participation in specific mechanisms by studying its possible partners. Previous preliminary evidence of the association between P2X7R and lamin A/C in chondrocyte-like cells such as IVD cells [34] suggested that this finding deserved to be explored in more details. Here we therefore extended the investigation to other nuclear proteins and employed additional methods, demonstrating for the first time the participation of P2X7R in molecular complexes crucial in the regulation of both nuclear structure maintenance and gene expression. Specifically, on the one hand, a close association between P2X7R and lamin A/C, SUN2, or emerin places this receptor within the context of factors involved in mechanosignaling and maintenance of nuclear integrity; on the other, its recruitment to promoters of specific gene suggests it is a transcription regulator. We are currently unable to provide a complete molecular map of how physical forces can influence biochemical responses through the function exerted by P2X7R in the molecular complexes in which it is present. However, we are confident that the P2X7R directly participates in changes occurring within a mechano-signal transduction pathway that begins in the plasma membrane and ends in the nucleus with modulations of gene transcription. Accordingly, based on what is known to date about the localization and roles of lamin A/C, SUN2, or emerin, P2X7R could be associated with nuclear pore complex, nuclear membrane and nucleoplasm [26, 55]. Several hypotheses regarding the interactions we have demonstrated deserve further investigation, as P2X7R could be a molecular component of several complexes that:

- mediate nucleocytoskeletal coupling of the lamina with the cytoskeleton via SUN domain proteins (LINC complexes that span the nuclear envelope), influencing both mechanosignal transduction and cell fate and differentiation;
- provide a structural scaffold for the efficient activation of cell signaling molecules based on the activity of specific phosphatases and kinases;
- regulate chromatin organization, including large chromatin domains called lamina-associated domains (LADs), transcription factor accessibility, and chromosomal tethering, contributing to the epigenetic regulation of genes during differentiation;
- recruit transcription factors, directly influencing gene transcription.

Most of these intriguing questions about the still undescribed functions of P2X7R will require confirmation through studies with genomic technologies including high-throughput methods, and experimental models suitable for responding to external stimuli such as mechanical loading applied to cells in combination with a 3D scaffold [56, 57]. Regarding the involvement of P2X7R in transcriptional regulation, this was first strengthened here through P2X7R overexpression experiments and ChIP assays conducted on human chondrocytes, further highlighting that P2X7R is not randomly located in the nucleus but has a potentially relevant role in chondrocyte behavior by contacting chromatin in regulatory regions, especially when cells are cultured in 3D. It is worth noting that the advantage of using a 3D cellular model lies primarily in providing a more natural environment for chondrocytes to: (i) produce a more adequate ECM compared to 2D culture; (ii) respond correctly to mechanotransduction based on cell-ECM communication and cell-ECM-cell crosstalk; and (iii) consequently, modulate gene expression through chromatin remodeling and epigenetic mechanisms. Although further experiments are needed, the findings from ChIP analysis provides clear evidence of a direct recruitment of P2X7R to regulatory regions belonging to specific genes. We can therefore state that, in the intracellular signaling context, P2X7R may also participate in a role different from that described in previous studies that have demonstrated the ability of P2X7R to activate transcription by triggering intracellular signaling pathways. This last aspect refers to certain transcription factors such as NF-κB, Sp1, AP-1, NFAT, HIF1α, STAT, and Egr 1–4, which can be activated by P2X7R-mediated actions through increased intracellular calcium levels, the formation of reactive oxygen species, the JAK or MAPK pathways, or the release of chemokines [5, 7, 41, 58]. Further investigations are needed to assess the biological relevance of the ChIP analysis results, since demonstrating that P2X7R is present in molecular complexes at regulatory regions in which lamin A/C binds is only a preliminary, albeit important, step. Considering that interactions, even weak ones, between proteins within molecular complexes can be critical for the control of gene expression, especially in response to environmental stimuli, we are confident that P2X7R may be involved in a regulatory context through transient associations or bindings whose strength may depend on the cell type and tissue microenvironment.

Overall, other important aspects that deserve further investigation concern the possible structural changes that P2X7R may undergo and that may affect its job, in particular its interaction with other proteins and consequently its control over chondrocyte responses. These include genetic variations, post-translational modifications, conformational changes during activation, or modulation by allosteric effectors, which together can influence its subcellular localization and activity.

In summary, this study highlights for the first time novel partners of P2X7R that place it into an intricate network that influences nuclear structure, mechanosensitivity, chromatin organization, and gene expression. Significant progress has been made in recent years in understanding the various aspects that characterize P2X7R biology, but filling the remaining gaps requires additional experimental approaches that are often difficult to apply. One such gap concerns the role of P2X7R in physiological contexts. We believe that the evidence gathered in this study can contribute in part to broadening our understanding of the relevance of this receptor in uninjured contexts such as normal chondrocytes. In the future, we can expect that methodological improvements, conceptual innovations, and the development of suitable human experimental models will accelerate our understanding of the physiological role of P2X7R in the signaling that supports the behavior of skeletal cells such as chondrocytes.

## Supplementary Information

Below is the link to the electronic supplementary material.


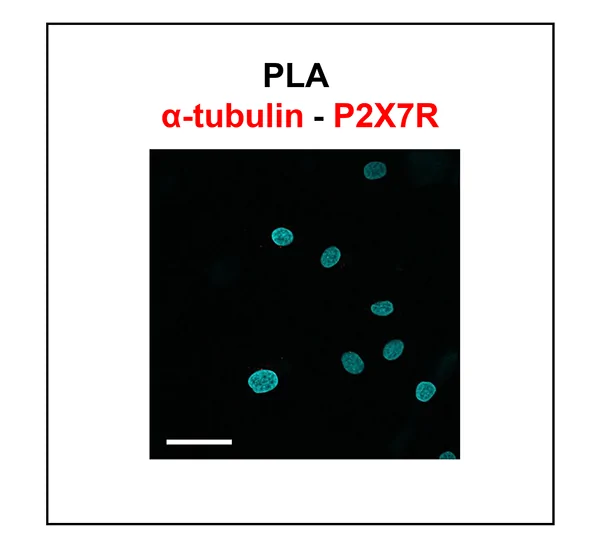




High Resolution(TIF 17.1 MB)



Supplementary Material 2(DOCX 3.33 MB)



Supplementary Material 3(DOCX 15.2 KB)


## Data Availability

No datasets were generated or analysed during the current study.

## Abbreviations

ChIP: Chromatin immunoprecipitation
Co-IP: Co-immunoprecipitation
Col2A1: Collagen Type II Alpha 1 Chain
CTGF: Connective tissue growth factor
Cx43: Connexin 43
DMEM: Dulbecco’s Modified Eagle Medium
eATP: Extracellular adenosine triphosphate
ECM: Extracellular matrix
FOXO3a: Forkhead box O3a transcription factor.
ITGB2: Integrin beta-2
LADs: Lamina-associated domains
LINC: Linker of Nucleoskeleton and Cytoskeleton
PLA: Proximity Ligation Assay.
P2X7R: Purinergic Receptor P2X7.
SOX9: SRY-box transcription factor 9
SUN2: SAD1/UNC84 domain protein-2
TEM: Transmission Electron Microscopy
TRPS1: Trichorhinophalangeal Syndrome 1 transcription factor
