# Supplementary material for: Participation of the purinergic P2X7 receptor in molecular complexes in the nucleus of human chondrocytes
Source: Cell Mol Life Sci. 2026 Jun 17;83(1):319. doi: 10.1007/s00018-026-06274-2 (PMC13534410; doi:10.1007/s00018-026-06274-2)
Supplement: Supplementary file 1 [file 18_2026_6274_MOESM2_ESM.docx]

**Supplementary Table 1** List of antibodies used in this study

| Antibody | Code | ICC dilution | IF dilution | WB dilution | PLA dilution | IP | ChIP |
| --- | --- | --- | --- | --- | --- | --- | --- |
| anti-lamin A/C | Santa Cruz, sc-7292 |  |  |  |  |  | 5 μg |
| anti-lamin A/C | Santa Cruz, sc-376248 |  | 1:3000 | 1:2000 | 1:3000 |  |  |
| anti-P2X7R | Alomone, APR-004 | 1:500 |  | 1:500 |  |  | 5 μg |
| anti-P2X7R | Merck, P9122 |  | 1:300 |  | 1:300 | 10 μg |  |
| anti-emerin | Monosan, MONX10804 |  | 1:200 | 1:500 | 1:200 |  |  |
| anti-SUN2 | Novus Biologicals, NBP2-59942 |  | 1:800 | 1:1000 | 1:800 |  |  |
| anti-α-tubulin | Santa Cruz, sc-23950 |  |  | 1:2500 | 1:500 |  |  |
| anti-β-actin | Santa Cruz, sc-8432 |  |  | 1:2500 |  |  |  |
| anti-COL2a1 | Abcam, ab3092 | 1:200 |  |  |  |  |  |
| anti-aggrecan | Santa Cruz, sc-33695 | 1:200 |  |  |  |  |  |
| anti-Sox9 | Abcam, AB5535 | 1:800 |  |  |  |  |  |
| anti-CD44 | Merck, MABF2092 | 1:200 |  |  |  |  |  |
|  |  |  |  |  |  |  |  |
| *Note. ChIP: chromatin immunoprecipitation assay; IF: immunofluorescence; IP: immunoprecipitation: PLA: proximity ligation assay; WB: western blot analysis.* | | | | | | | |
