# Supplementary material for: Participation of the purinergic P2X7 receptor in molecular complexes in the nucleus of human chondrocytes
Source: Cell Mol Life Sci. 2026 Jun 17;83(1):319. doi: 10.1007/s00018-026-06274-2 (PMC13534410; doi:10.1007/s00018-026-06274-2)
Supplement: Supplementary file 2 [file 18_2026_6274_MOESM3_ESM.docx]

**Supplementary Table 2**  List of primers used in this study

| Primers | Forward Sequence (5′−3′) | Reverse Sequence (5′−3′) |
| --- | --- | --- |
| **Sox9**  Upstream  Downstream | TGAGAGACACCACCAATGCC  ACACAATGCCCTTCTACCCG | GCCGGTGCTTCTGACTCTAG  TCGTATCCAGGGCTCTGTGA |
| **TRPS1**  Upstream  Downstream | GGTACCCCCTTAAAGCACCC  TGGAAATTGGTGGTGGACAGT | GTTGGAGAAGTGCGGACTGA  AGTACTGCATGCCGGGAAAA |
| **FOXO3a**  Upstream  Downstream | TCCTCTCCTCCCTTCTCC  GTGCGCTGCAACTTTCCTC | CGAGAGTCTCAACGACAACA  GAGGGAAAAAGGGAGAGGGC |
| **CTGF**  Upstream  Downstream | CAGTGGACAGAACAGGGCAA  CCTTTGCTGGCCATTCACAC | TCCCTATCCTCTTCGCACCA  TCGTTAAGCCAGCCACATGT |
| **ITGB2**  Upstream  Downstream | TCCTCCTGTGACCTGAGTGT  AGATGAGGGTCTGGGTCTCC | ACATCCACCTGCATGTGAGG  GGCATGGTGGCTCAAACTTG |
| **Cx43**  Upstream | GCAACTCCAAGTAGAGGCGT | GGTAGGGAGGAGGCTGGTAA |
